# Evaluation of coercive measures in different psychiatric hospitals: the impact of institutional characteristics

**DOI:** 10.1186/s12888-021-03410-z

**Published:** 2021-08-21

**Authors:** Klaus Mann, Sonja Gröschel, Susanne Singer, Jörg Breitmaier, Sylvia Claus, Markus Fani, Stephan Rambach, Hans-Joachim Salize, Klaus Lieb

**Affiliations:** 1 Department of Psychiatry and Psychotherapy, University Medical Center, Untere Zahlbacher Str. 8, 55131 Mainz, Germany; 2 Department of Neurology, University Medical Center, Mainz, Germany; 3 Institute of Medical Biostatistics, Epidemiology and Informatics, University Medical Center, Mainz, Germany; 4 Department of Psychiatry and Psychotherapy, Krankenhaus Zum Guten Hirten, Ludwigshafen, Germany; 5 Department of Psychiatry, Psychosomatics und Psychotherapy, Pfalzklinikum, Klingenmünster, Germany; 6 Department of Geriatric Psychiatry, Psychosomatics und Psychotherapy, Pfalzklinikum, Klingenmünster, Germany; 7 Clinic for Psychiatry and Psychotherapy, Municipal Hospital, Pirmasens, Germany; 8 Central Institute of Mental Health, Medical Faculty Mannheim / Heidelberg University, Mannheim, Germany

**Keywords:** Coercive measures, Organization of psychiatric inpatient care, Safety, Patient autonomy

## Abstract

**Background:**

Epidemiological studies have demonstrated considerable differences in the use of coercive measures among psychiatric hospitals; however, the underlying reasons for these differences are largely unclear. We investigated to what extent these differences could be explained by institutional factors.

**Methods:**

Four psychiatric hospitals with identical responsibilities within the mental health care system, but with different inpatient care organizations, participated in this prospective observational study. We included all patients admitted over a period of 24 months who were affected by mechanical restraint, seclusion, or compulsory medication. In addition to the patterns of coercive measures, we investigated the effect of each hospital on the frequency of compulsory medication and the cumulative duration of mechanical restraint and seclusion, using multivariate binary logistic regression. To compare the two outcomes between hospitals, odds ratios (OR) with corresponding 95% confidence intervals (CI) were calculated.

**Results:**

Altogether, coercive measures were applied in 1542 cases, corresponding to an overall prevalence of 8%. The frequency and patterns of the modalities of coercive measures were different between hospitals, and the differences could be at least partially related to institutional characteristics. For the two hospitals that had no permanently locked wards, certain findings were particularly noticeable. In one of these hospitals, the probability of receiving compulsory medication was significantly higher compared with the other institutions (OR 1.9, CI 1.1–3.0 for patients < 65 years; OR 8.0, CI 3.1–20.7 for patients ≥65 years); in the other hospital, in patients younger than 65 years, the cumulative duration of restraint and seclusion was significantly longer compared with the other institutions (OR 2.6, CI 1.7–3.9).

**Conclusions:**

The findings are compatible with the hypothesis that more open settings are associated with a more extensive use of coercion. However, due to numerous influencing factors, these results should be interpreted with caution. In view of the relevance of this issue, further research is needed for a deeper understanding of the reasons underlying the differences among hospitals.

## Background

Coercive measures in the form of mechanical restraint, seclusion, and compulsory administration of medication are widely used in psychiatric institutions for the management of patient behaviors that are potentially harmful to themselves or to others. Due to the ethical and legal compliance issues surrounding the conflict between ensuring safety versus respecting patient autonomy, the adequate application of coercive measures represents major challenges in everyday clinical practice [1–6].

There is broad consensus that coercive measures should only be used as a last resort when the hazard posed by the patient’s behavior cannot be otherwise controlled [7, 8]. However, there is still a significant lack of empirical data regarding the benefits and risks associated with both coercive measures and potential alternatives, despite the proposal of various strategies to reduce the use of coercion [8–13]. Of note, epidemiological studies have demonstrated considerable differences in the frequency and methods of coercive measures between individual institutions within various countries [13–19]. Overall, clinical practice appears to be primarily determined by local traditions and personal preferences instead of by evidence. Thus, there is a tremendous need to examine the inconsistencies in the use of coercion aimed at reducing the total number of coercive measures and, for cases in which coercion is unavoidable, choosing the most effective yet safe and humane method as possible.

The use of coercive measures is determined by numerous influencing factors. In addition to patient characteristics and the competencies and attitudes of the staff, structural and organizational aspects of inpatient care play important roles [20–25]. In this context, the concept of “open-door psychiatry” is increasingly being discussed, but the subject is controversial. Although locked wards may be necessary to ensure safety, locked doors may lead to more frequent critical incidents and an increased need for coercion due to their aversive nature. In fact, some empirical data indicate that open-door policies are not associated with more frequent complications [26–29]. However, given that considerable methodological objections have been raised against these studies, no clear recommendations can be made at this time [6, 8, 30–32]. In particular, the effects of open-door versus locked-ward policies on the use of coercive measures are unclear.

Against this background, we assessed the application of coercive measures in psychiatric inpatients treated under real care conditions in different hospitals. The primary objective was to investigate, on an exploratory basis, whether there were differences in the use of coercive measures between participating hospitals, and to what extent these differences could be explained by the organization of inpatient care within the hospitals, including the impact of differing door policies.

## Methods

In a multicenter prospective observational study, we enrolled patients in 4 psychiatric hospitals located in the German federal state of Rhineland-Palatinate over a period of 24 months. The hospitals were selected in such a way that, on the one hand, there were clear differences with regard to institutional characteristics, whereas on the other hand, they had identical care mandates.

### Participating institutions

All hospitals are included in the governmental hospital planning headed by the State Ministry of Health and have the legal obligation to take over comprehensive psychiatric inpatient care, including emergency hospitalizations, for a defined catchment area. Rhineland-Palatinate is divided into 19 care regions, in each of which one hospital has a mandatory care mandate; the catchment areas of the 4 participating hospitals cover 23% of the total population of this federal state.

Against a background of identical duties and responsibilities within the mental health care system, there are clear differences in structure, organization, and patient management:

- Hospital A is a specialty hospital for psychiatry and neurology with a predominantly rural catchment area (population 399,000, area 1373 km^2^). This is a teaching hospital of a university medical center. Two departments participated in the study:
  - The Department of General Psychiatry (patients < 65 years of age) with 166 beds on 6 open wards and 2 locked wards. All wards are specialized in regards to diagnoses.
  - The Department of Geriatric Psychiatry (patients ≥65 years of age) with 61 beds on 1 open ward and 2 locked wards. These wards are not specialized in terms of diagnoses.
  In relation to the catchment area, the two departments provide 0.57 beds per 1000 inhabitants. Critical patients who exhibit a risk potential are initially admitted to one of the locked wards. Coercive measures are carried out only on the locked wards. Patients are transferred between open and locked wards if necessary and justifiable on the basis of the risk assessment.

- Hospital B is a department at a nonacademic general hospital with an urban catchment area (population 172,000, area 78 km^2^). This hospital has 74 beds on 3 open wards (0.43 beds per 1000 inhabitants). The ward doors are principally open, but can be locked if necessary. The wards are not specialized in terms of diagnoses. Admissions are made regardless of the place of residence in the catchment area; however, in order to optimize the continuity of treatment, efforts are made to treat readmitted patients on the same ward as they were on previously. Critical inpatients with risk potential are treated on all wards, and coercive measures are also used on all wards.

- Hospital C is a department at a university medical center with an urban catchment area (population 219,000, area 98 km^2^). This hospital has 125 beds on 6 open wards and 1 locked ward (0.57 beds per 1000 inhabitants). Whereas patients on the locked ward have the full spectrum of diagnoses, the open wards are specialized by diagnosis. The one ward specializing in geriatric psychiatric patients basically has an open ward door, although it can be locked if necessary. Critical patients with risk potential are initially admitted to the locked ward, whereas the other patients are admitted to the other wards according to diagnosis. Coercive measures are predominantly carried out on the locked ward and only a small proportion are performed on the geriatric ward. Patients are transferred between the open and locked wards if necessary and justifiable on the basis of the risk assessment.

- Hospital D is a department at a nonacademic general hospital with a catchment area containing both urban and rural sectors (population 169,000, area 1086 km^2^). This hospital has 80 beds on 3 open wards (0.47 beds per 1000 inhabitants). The hospital leadership advocates a strict open-door policy: the ward doors are open at all times and cannot be locked, but are under intensive surveillance by staff. Admissions are made according to the principle of sectorization, i.e., each ward is assigned to a specific sector of the catchment area. The wards are not specialized in terms of diagnoses. Critical inpatients with risk potential are treated on all wards, and coercive measures are also used on all wards.

In each of the facilities described above, all wards participated in the study. Thus, it was ensured that all institutions relevant for inpatient care of the adult population in the 4 assigned catchment areas were involved. Due to very different treatment conditions and legal frameworks, the departments for child and adolescent psychiatry in hospital A and at the university medical center, to which hospital C belongs, as well as the department for forensic psychiatry in hospital A, were not involved. Although coercive measures are also used in these facilities, they were not relevant to the objectives of the present study.

In the statistical analysis where we compared the hospitals regarding the use of coercive measures, the organizational characteristics of the hospitals described above represent predetermined and fixed parameters.

### Study sample

All patients admitted to one of the 4 hospitals during the recruitment period from October 1, 2012, to September 30, 2014, who experienced one or more of the following coercive measures during the course of the index hospitalization, were included in the study: mechanical restraint of variable extent in a bed or chair, seclusion in a special room, or compulsory administration of medication. All forms of forced medication were considered, regardless of the type of substance and indication. There were no further inclusion or exclusion criteria.

The study was performed under real care conditions with no study-related interventions. All patients were treated according to standard procedures in the respective hospitals. No study-specific treatment requirements or recommendations were made. The safety and quality of care were exclusively incumbent upon the institutions and were explicitly not influenced by the study design.

### Data collection

In addition to the usual patient charts, a standardized form was used to document the coercive measures. This form had already been implemented in routine care in all the participating hospitals before the study began. The coercive measures data as well as the sociodemographic and basic clinical data were extracted from the patient charts by a member of the project team during regularly scheduled visits at participating hospitals. All the data were entered in electronic devices in the respective hospitals and were sent in a pseudonymous form to the study center in Mainz for subsequent analysis. In the participating hospitals, all admissions are fully recorded administratively, so that the total number of admissions during the study period can be obtained from the respective clinical information systems.

Documentation quality was ensured by quality safety measures already in place at the various institutions under the responsibility of the local chief physicians. Moreover, the quality and completeness of the extracted data were continuously monitored by the project team in parallel with data collection during the entire study period.

### Data analysis

A statistical analysis plan was drafted by the data analyst based on the original grant proposal and subsequently discussed with the principal investigator and all authors. Several revisions of this plan were performed. The final version of the analysis plan was used for the following pre-planned data analyses. No post-hoc analyses were conducted.

The data analyst was blinded with respect to the hospitals and was only aware of the hospitals’ pseudonyms (A, B, C, and D). The prevalence of coercive measures was calculated by the number of cases affected by coercion in relation to the total number of admissions in each hospital. A more detailed evaluation of the differences in relative frequencies of the use of coercive measures among the hospitals was not performed because the characteristics of those patients who were not affected by coercive measures were not recorded in this study. Instead, we focused on how coercive measures were applied in the clinical routine and whether differences between hospitals could be related to structural and organizational factors.

In the first step, we analyzed patterns of coercive measures applied in the various hospitals. For each case during their entire hospital stay, distinct episodes of coercion were defined as continuing mechanical restraint or seclusion, including interruptions of less than 6 h. Interruptions longer than 6 h and changes from mechanical restraint to seclusion or vice versa were considered separate episodes of coercion. The duration of each episode was calculated by summarizing the entire duration minus the time of interruptions (i.e., the net duration). Next, the cumulative duration of mechanical restraint and seclusion per case was calculated by summing all episodes’ net duration. Given the cumulative duration was not normally distributed, we dichotomized it into ≤8 and > 8 h. Compulsory medication could be administered alone without further coercive measures or in combination with mechanical restraint or seclusion. Sample characteristics (sex, age, and ICD-10 F diagnosis) were compared between the hospitals using percentages and chi-square tests.

In the second step, we investigated the hospital effects on two outcomes relevant to the application of coercive measures using multivariate binary logistic regression: frequency of compulsory administration of medication (cases with vs. without compulsory medication) and cumulative duration of mechanical restraint and seclusion per case (≤ 8 vs. > 8 h).

Effect modification by age (≥ 65 and <  65 years) was anticipated a priori and was tested using likelihood ratio tests. In both regression models, there was indeed evidence of effect modification by age. Hence, analyses were performed separately for the two age groups. To compare the two outcomes between hospitals, odds ratios (OR) with corresponding 95% confidence intervals (CI) were calculated, adjusting for potential confounding variables (patients’ sex and psychiatric diagnosis, occupancy per staff, and modality of the coercive measure). In the regression models, we used hospital A as the reference because it comprised the largest sample size. Hence, the OR corresponds to the likelihood (odds) of receiving compulsory medication and having a cumulative duration of mechanical restraint and seclusion per case of > 8 h, respectively, in the hospitals B, C, and D compared with hospital A.

We performed the statistical analysis using the STATA 12 software package (StataCorp 2011, College Station, TX: StataCorp LP).

### Ethical and legal considerations

The study protocol was approved by the ethics committee at the state chamber of physicians of Rhineland-Palatinate, reference number 837.515.11 (8057). Regarding the legal aspects of the use of coercive measures, unitary statutory regulations and medical guidelines were followed at the participating hospitals.

## Results

Altogether, we registered 1542 cases that were affected by coercive measures, corresponding to an average proportion of 8.0% of all cases admitted to the participating hospitals during the 2-year recruitment period. In relation to the total number of admissions in each hospital, the proportion of cases affected by coercive measures was 5.2% for hospital A, 9.9% for hospital B, 9.4% for hospital C, and 11.7% for hospital D.

### Characterization of the study sample

The patients’ demographic and clinical characteristics are summarized in Table 1. There were no differences in sex distribution between the hospitals. The average percentage of cases aged ≥65 years was 28%. In hospitals A and D, the proportion of elderly patients was higher compared with that in hospitals B and C. On the basis of the ICD-10 diagnoses, substance-related disorders (F1) were most frequent, followed by organic mental disorders (F0) and schizophrenia and other psychotic disorders (F2). F0 diagnoses were more frequent in hospitals A and D, whereas F1 and F2 diagnoses were more frequent in hospitals B and C.

**Table 1:** Characteristics of cases affected by coercive measures

	All	Hosp A	Hosp B	Hosp C	Hosp D	
	n (%)	n (%)	n (%)	n (%)	n (%)	*P* value
Admissions	19,295	8571	4268	3505	2951	
Number of cases with coercive measures	1542	448	422	328	344	
Sex						
Female	533 (35)	149 (33)	149 (35)	116 (35)	119 (35)	0.91
Male	1009 (65)	299 (67)	273 (65)	212 (65)	225 (65)	
Age						
< 65 years	1105 (72)	277 (62)	353 (84)	259 (79)	216 (63)	< 0.01
≥ 65 years	437 (28)	171 (38)	69 (16)	69 (21)	128 (37)	
Diagnosis ICD-10						
F0	419 (27)	177 (40)	57 (14)	66 (20)	119 (35)	< 0.001
F1	523 (34)	98 (22)	183 (43)	131 (40)	111 (32)	< 0.001
F2	365 (24)	87 (19)	134 (32)	72 (22)	72 (21)	< 0.001
F3	94 (6)	26 (6)	18 (4)	37 (11)	13 (4)	< 0.001
F4/5	29 (2)	13 (3)	11 (3)	4 (1)	1 (0)	0.03
F6	56 (4)	23 (5)	12 (3)	18 (5)	3 (1)	0.002
F7/8	56 (4)	24 (5)	7 (2)	0 (0)	25 (7)	< 0.001

*Abbreviations: Hosp* Hospital

### Pattern of coercive measures

**Fig. 1:**
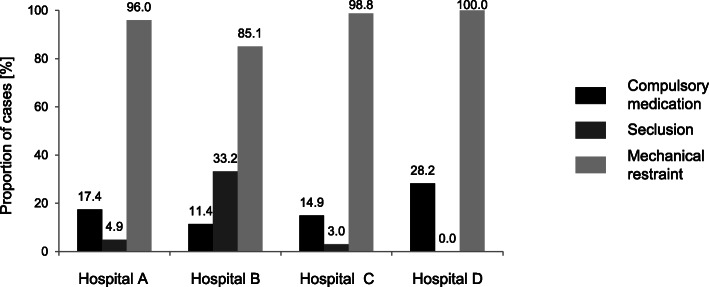
Proportion of cases [%] of compulsory medication, seclusion, and mechanical restraint in the participating hospitals (total number of cases 1542)

Of all 1542 cases, in 1200 cases (77.8%) only one distinct coercive modality was applied; in the remaining cases, more than one method was used during the patient’s stay in the hospital. The proportion of cases affected by the distinct coercive modalities in the various hospitals is shown in Fig. 1. In all hospitals, mechanical restraint was the predominant method when applying coercive measures. In hospital D, all patients who underwent coercion had mechanical restraints, and no seclusions were performed. The percentage of cases concerned by seclusion was the highest in hospital B (33.2%), whereas it was low in hospitals A and C (< 5%). Overall, medication was compulsorily administered in 272 cases (17.6%). The highest proportion was recorded in hospital D (28.2%), whereas the lowest was found in hospital B (11.4%). Compulsory medication was primarily administered in combination with mechanical restraint.

The number of distinct episodes of coercion within an individual case ranged from 1 to 21. The majority of the patients (71.9%) underwent only one episode of coercion during their hospital stay, and in most cases (72.0%) the first episode occurred on the day of admission or the day after admission.

### Compulsory administration of medication

**Fig. 2:**
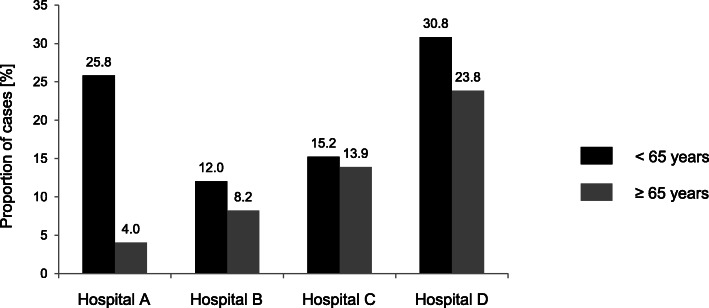
Proportion of cases [%] of compulsory medication in different hospitals depending on age

In all the hospitals, the proportion of cases of compulsory medication was higher in younger patients compared with patients aged ≥65 years (Fig. 2).

The statistical analysis of the differences in administration of compulsory medication between hospitals is summarized in Table 2. Unadjusted ORs are shown in the upper part of the table. After adjusting for sex, diagnosis, and occupancy per staff, only slight changes in the ORs were observed. However, an independent effect of these variables on the administration of compulsory medication was evident. For hospital D, the probability of receiving compulsory medication was higher compared with that for hospital A for both age groups (age < 65 years, OR 1.9 (1.1,3.0); age ≥ 65 years, OR 8.0 (3.1,20.7)). For hospitals B and C, the probability of receiving compulsory medication was lower in younger patients compared with that for hospital A (OR 0.5 (0.3,0.8)). By contrast, for elderly patients, the probability of receiving compulsory medication was higher in hospital C (OR 5.2 (1.7,15.8)) and tended to be higher in hospital B compared with hospital A.

**Table 2:** Probability of receiving compulsory medication, stratified by age, unadjusted and adjusted for diagnosis, sex, and occupancy per staff

	< 65 years		≥ 65 years	
	OR (95% CI)	*P* value	OR (95% CI)	*P* value
Hosp A	reference		reference	
Hosp B	0.4 (0.3,0.6)	< 0.01	2.2 (0.7,6.9)	0.16
Hosp C	0.5 (0.3,0.8)	< 0.01	4.0 (1.4,10.9)	0.01
Hosp D	1.3 (0.9,1.9)	0.23	7.5 (3.2,17.7)	< 0.01
Hosp A	reference		reference	
Hosp B	0.5 (0.3,0.8)	0.01	2.5 (0.7,8.5)	0.15
Hosp C	0.5 (0.3,0.8)	< 0.01	5.2 (1.7,15.8)	< 0.01
Hosp D	1.9 (1.1,3.0)	0.01	8.0 (3.1,20.7)	< 0.01
Diagnosis ICD-10				
F0	reference		reference	
F1	1.6 (0.7,3.7)	0.29	0.8 (0.2,2.8)	0.71
F2	4.0 (1.7,9.3)	< 0.01	4.9 (2.0,12.2)	< 0.01
F3	3.8 (1.5,9.9)	0.01	0.6 (0.1,4.5)	0.58
F4/5/6	2.7 (1.0,7.0)	0.05	39.8 (2.8,575.0)	0.01
F7/8	1.4 (0.5,4.0)	0.53	omitted	
Male sex	0.7 (0.5,0.9)	0.02	0.8 (0.4,1.5)	0.45
Patients per staff	0.9 (0.9,1.0)	0.02	1.0 (0.9,1.2)	0.46

*Abbreviations*: *Hosp* Hospital, *OR* Odds ratio, *CI* Confidence interval

Regarding diagnoses, schizophrenia and other psychotic disorders (ICD-10: F2) were associated with a higher probability of receiving compulsory medication compared with organic mental disorders (ICD-10: F0) and substance use disorders (ICD-10: F1).

### Cumulative duration of mechanical restraint and seclusion per case

**Fig. 3:**
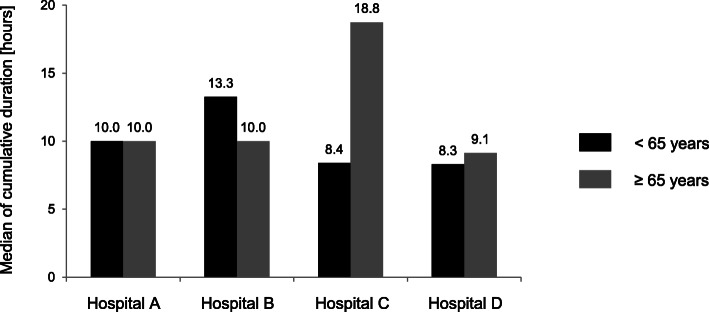
Median of cumulative duration of mechanical restraint and seclusion per case [hours] in different hospitals depending on age

The cumulative duration of all motion-restricting coercive measures shows a markedly skewed distribution with a wide range (median 10.2 h, mean 42.7 h). Clear differences between hospitals were evident, and these relationships were different between the two age groups (Fig. 3).

The statistical analysis of the cumulative duration is summarized in Table 3. Unadjusted ORs are shown in the upper part of the table. The ORs did not change considerably after adjusting for sex, diagnosis, and occupancy per staff. In the younger patients, the probability of a cumulative duration > 8 h was higher in hospital B compared with hospital A (OR 3.4 (2.3,5.1)). For elderly patients, however, the probability was higher in hospital C compared with hospital A (OR 2.7 (1.4,5.5)). In the younger age group, the diagnosis of substance use disorder (ICD-10: F1) was associated with a lower probability of a cumulative duration > 8 h compared with other diagnoses, and male patients were more likely to experience a cumulative duration > 8 h than female patients.

**Table 3:** Probability that the cumulative duration of mechanical restraint and seclusion per case is > 8 h, stratified by age, unadjusted and adjusted for given variables

	< 65 years		≥ 65 years	
	OR (95% CI)	*P* value	OR (95% CI)	*P* value
Hosp A	reference		reference	
Hosp B	2.1 (1.5,3.0)	< 0.01	0.9 (0.5,1.6)	0.75
Hosp C	0.8 (0.6,1.1)	0.25	2.9 (1.5,5.7)	< 0.01
Hosp D	0.8 (0.5,1.1)	0.18	0.9 (0.6,1.4)	0.65
Hosp A	reference		reference	
Hosp B	3.4 (2.3,5.1)	< 0.01	1.1 (0.6,2.0)	0.83
Hosp C	1.0 (0.7,1.5)	0.95	2.7 (1.4,5.5)	< 0.01
Hosp D	1.1 (0.7,1.6)	0.75	1.0 (0.6,1.7)	0.87
Diagnosis ICD-10				
F0	reference		reference	
F1	0.2 (0.1,0.5)	< 0.01	0.6 (0.3,1.3)	0.19
F2	0.8 (0.4,1.5)	0.52	0.9 (0.4,2.0)	0.87
F3	0.8 (0.4,1.8)	0.65	2.1 (0.7,6.9)	0.21
F4/5/6	0.8 (0.4,1.7)	0.54	omitted	
F7/8	1.4 (0.6,3.2)	0.47	omitted	
Male sex	1.4 (1.1,1.9)	0.01	1.5 (1.0,2.3)	0.04
Patients per staff	1.0 (0.9,1.0)	0.07	0.9 (0.9,1.0)	0.13
Hosp A	reference		reference	
Hosp B	2.6 (1.7,3.9)	< 0.01	0.7 (0.4,1.5)	0.38
Hosp C	1.1 (0.7,1.5)	0.79	2.7 (1.4,5.5)	< 0.01
Hosp D	1.1 (0.7,1.7)	0.55	1.0 (0.6,1.7)	0.99
Diagnosis ICD-10				
F0	reference		reference	
F1	0.3 (0.2,0.5)	< 0.01	0.7 (0.3,1.4)	0.29
F2	0.7 (0.4,1.4)	0.32	0.8 (0.4,1.9)	0.66
F3	0.9 (0.4,1.8)	0.70	1.7 (0.5,5.9)	0.37
F4/5/6	0.8 (0.4,1.7)	0.62	omitted	
F7/8	1.3 (0.6,3.2)	0.50	omitted	
Male sex	1.5 (1.1,2.0)	0.01	1.5 (1.0,2.3)	0.05
Patients per staff	1.0 (0.9,1.0)	0.11	1.0 (0.9,1.0)	0.22
Compulsory medication	1.0 (0.7,1.4)	0.88	1.2 (0.6,2.2)	0.67
Seclusion	4.8 (2.5,9.1)	< 0.01	5.2 (1.3,20.6)	0.02

*Abbreviations*: *Hosp* Hospital, *OR* Odds ratio, *CI* Confidence interval

Finally, the effects of the compulsory medication administration and the modality of coercive measures (mechanical restraint versus seclusion) were included as additional variables. There was no evidence of an independent effect of compulsory medication on the cumulative duration. However, seclusion was associated with a higher probability of a cumulative duration > 8 h. The effect of the hospital was reduced for hospital B, where the OR changed from 3.4 to 2.6 for patients younger than 65 years. However, even after adjusting for the effect of seclusion, a higher probability of a cumulative duration > 8 h persisted in hospital B compared with the other hospitals.

## Discussion

We found a high overall prevalence of coercive measures of 8% in the participating hospitals, which concurs with previous studies [16, 33, 34]. A more detailed evaluation of the differences in relative frequencies of coercive measures among the hospitals was not performed because the characteristics of those patients who were not affected by coercive measures were not recorded in this study. Instead, we focused on how coercive measures were applied in the clinical routine.

The first focus of the study was on the frequency of compulsory medication. Across all hospitals, compulsory medication was less frequently administered to elderly patients. This could be due to several reasons, including differing indications for coercive measures, e.g., prevention of falls predominately in elderly individuals, and a more cautious administration of medication in case of organic mental disorders or somatic comorbidities. Particularly remarkable is the low proportion of compulsory medication in the older age group in hospital A compared with the other hospitals. This difference could be related to the strict organizational segregation of the general psychiatric and geriatric psychiatric inpatients at this hospital. Segregation allows a high degree of specialization, particularly in the geriatric psychiatric department, which is characterized by special competence and experience in dealing with elderly individuals and their age-specific problems [35–38]. However, the advantages of specialization must be weighed against its possible negative effects such as the marginalization of the elderly and the one-sided burden on the staff [39].

A relatively high proportion of cases of compulsory medication use was found in hospital D regardless of age. We hypothesize that this could be at least partially due to the structural aspects of this hospital. A lack of seclusion capabilities in association with open, non-lockable wards might require alternative coercive actions, including compulsory medication, to keep the duration of mechanical restraint short while limiting the risk of absconding from the ward. In all the other hospitals, the patients could be secluded (e.g., immediately after mechanical restraint) or treated under locked-ward conditions.

However, alternative explanations must also be considered. A key issue is the attitude of the staff members concerning the administration of compulsory medication [40–45]. Within the legal framework, there is a certain scope for decision-making between avoiding compulsory medication as far as possible, because this can be regarded as an additional somatically invasive coercive measure accompanied by further risks, and administering compulsory medication as soon as this can be justified to mitigate symptoms and shorten motion-restricting measures. These individual preferences could vary between hospitals and thus could contribute to the differences observed.

The other critical parameter we focused on was the cumulative duration of mechanical restraint and seclusion. Of note, compulsory medication had no significant influence on the cumulative duration. One explanation could be that compulsory medication was preferred in a subgroup of patients who were particularly severely disturbed, and the forced medication could have shortened the duration of restricted mobility that would otherwise have been much longer. However, as a relevant confounding variable, seclusion instead of mechanical restraint was identified. A possible explanation for the association between seclusion and a longer cumulative duration could be that seclusion was perceived by the staff as less invasive compared with mechanical restraint, which might tempt the staff to maintain it longer. This possibility is suggested by empirical findings that patients predominantly perceive seclusion as less stressful than mechanical restraint [46–49]. In this context, it is worth mentioning a previous study which had shown that compulsory medication was perceived by patients as less stressful than mechanical restraint and seclusion [50].

The interaction between hospital and age regarding the cumulative duration indicates different practices in the hospitals for younger and elderly patients. In the younger age group, a longer duration was more likely in hospital B compared with the other hospitals. This difference could be at least partially due to the higher proportion of seclusion cases in this hospital. In addition, we hypothesize that longer durations could be facilitated by the structural conditions in hospital B, which has open wards that can be locked if required. Avoidance of locking could reinforce the tendency to prolong mechanical restraint and seclusion compared with settings that have continuously locked wards, such as in hospitals A and C. In hospital D, which has permanently open wards, the staff might be more aware of the risk of absconding. Moreover, the relatively high proportion of compulsory medication could also reduce the risk of absconding in that hospital, as discussed earlier.

However, other influencing factors should also be considered here. In particular, the attitudes of the staff members with respect to mechanical restraint and seclusion play a crucial role and can differ among the hospitals [51–55]. Here, the key focus is on the area of conflict between the staff’s risk-taking propensity and their respect for patient autonomy. This trade-off essentially determines not only the indication for the use of coercion but also the decision regarding its termination: a higher need for safety on the part of the staff and less respect for the patient’s right to self-determination will be associated with a longer duration of coercive measures.

In older patients, the probability of a longer duration was increased in hospital C compared with the other hospitals. Structural aspects cannot be clearly related to this, so staff attitudes and their methods of dealing with specific risks in geriatric psychiatric patients might explain this finding. To reveal the underlying causes, a more in-depth analysis would be required to assess the medical and nursing approaches to the care of elderly patients.

The findings in this study are compatible with the hypothesis that open-door settings could increase the use of compulsory medication or prolong the duration of motion restraint. However, interpretations regarding the impact of structural aspects must be made with caution. One reason is the ethical issues already discussed earlier with complex trade-offs between conflicting values. The individual attitudes and preferences concerning these issues are predetermined by personality traits and vocational experiences and are shaped by institutional culture. Moreover, decision-making regarding the application of coercive measures is influenced by group processes, such as communication and dealing with emotional stress within the team. These aspects have a decisive impact on the way critical situations are handled [56–60], which might be different in hospitals, but they are very complex and have not been investigated in this study.

There are also other limitations to this study. One relevant factor is the severity of the behavioral disturbances and the resultant hazard potential, which has not been assessed in the present study [61, 62]. Thus, patients in hospitals with higher rates of coercive medication or a longer duration of mechanical restraint and seclusion could have been more severely disturbed compared with the patients in other hospitals. The reasons for such regional differences in severity could be related to differences in sociodemographic characteristics or the structure of complementary facilities in the respective catchment areas.

Furthermore, we did not consider the use of alternative means to ensure patient and staff safety that excludes mechanical restraint or seclusion, e.g., installing a door guard or intensive one-on-one care of high-risk patients. However, the patients might also consider such alternative approaches to be very restrictive. Finally, we only assessed the impact of the structural aspects on the implementation of coercive measures, and we did not investigate the impact of these structural aspects on the prevention of critical incidents and subsequent coercive measures. In this respect, the opinion is often expressed that open-door policies have a de-escalating effect, resulting in a reduction in critical situations. However, considering the last two points would require different study designs, in which cases not affected by coercive measures are also studied, including the monitoring of the complications that have occurred under these conditions.

## Conclusions

This study confirms the clear differences in the use of coercive measures between psychiatric hospitals, which could be related at least in part to specific institutional characteristics. From our findings, the hypothesis can be formulated that more open settings are associated with a more extensive use of coercion in the form of compulsory medication or a longer duration of motion-restricting measures. However, due to the many factors influencing the use of coercive measures, the results should be interpreted with caution. Given the significant relevance of the topic for the organization of psychiatric inpatient care, further research is needed for a deeper understanding of the reasons underlying the differences between hospitals.

From the clinical perspective, in addition to the efforts within each institution, a regular exchange of experiences between staff members at different hospitals in the context of clinical routine could promote mutual learning processes to optimize the handling of critical incidents and the implementation of coercive measures in psychiatric institutions.

## Data Availability

All details regarding study design, data collection, and data analysis will be provided by the corresponding author upon request.

## Abbreviations

ICD-10: International Classification of Diseases, 10th revision
OR: Odds ratio
CI: Confidence interval
Hosp: Hospital

## References

[1] Kallert TW (2008). Coercion in psychiatry. Curr Opin Psychiatry.

[2] Prinsen EJD, van Delden JJM (2009). Can we justify eliminating coercive measures in psychiatry?. J Med Ethics.

[3] Perkins E, Prosser H, Riley D, Whittington R (2012). Physical restraint in a therapeutic setting: a necessary evil?. Int J Law Psychiatry.

[4] Hem MH, Gjerberg E, Husum TL, Pedersen R (2018). Ethical challenges when using coercion in mental healthcare: a systematic literature review. Nurs Ethics.

[5] Haugom EW, Ruud T, Hynnekleiv T (2019). Ethical challenges of seclusion in psychiatric inpatient wards: a qualitative study of the experiences of Norwegian mental health professionals. BMC Health Serv Res.

[6] Pollmächer T (2019). Autonomy focusing as guiding idea of minimally restrictive psychiatry. Nervenarzt..

[7] National Institute for Health and Care Excellence (NICE) (2015). Violence and aggression: short-term management in mental health, health and community settings.

[8] Deutsche Gesellschaft für Psychiatrie und Psychotherapie, Psychosomatik und Nervenheilkunde (DGPPN). S3-Leitlinie Verhinderung von Zwang: Prävention und Therapie aggressiven Verhaltens bei Erwachsenen. 2018. https://www.dgppn.de/leitlinien-publikationen/leitlinien.html. Accessed 11 Mar 2021.

[9] Muralidharan S, Fenton M. Containment strategies for people with serious mental illness. Cochrane Database Syst Rev. 2006;(3):CD002084. 10.1002/14651858.CD002084.pub2. 10.1002/14651858.CD002084.pub2 PMC11384505 16855984

[10] Nelstrop L, Chandler-Oatts J, Bingley W, Bleetman T, Corr F, Cronin-Davis J (2006). A systematic review of the safety and effectiveness of restraint and seclusion as interventions for the short-term management of violence in adult psychiatric inpatient settings and emergency departments. Worldviews Evid-Based Nurs.

[11] Hirsch S, Steinert T (2019). Measures to avoid coercion in psychiatry and their efficacy. Dtsch Arztebl Int.

[12] De Bruijn W, Daams JG, van Hunnik FJG, Arends AJ, Boelens AM, Bosnak EM, et al. Physical restraints in hospital care: protocol for a systematic review. Front Psychiatry. 2020;10:921. 10.3389/fpsyt.2019.00921. 10.3389/fpsyt.2019.00921 PMC7058582 32184738

[13] Sashidharan SP, Mezzina R, Puras D (2019). Reducing coercion in mental healthcare. Epidemiol Psychiatr Sci.

[14] Way BB, Banks SM (1990). Use of seclusion and restraint in public psychiatric hospitals: patient characteristics and facility effects. Hosp Community Psychiatry.

[15] Ray NK, Rappaport ME (1995). Use of restraint and seclusion in psychiatric settings in New York state. Psychiatr Serv.

[16] Steinert T, Martin V, Baur M, Bohnet U, Goebel R, Hermelink G (2007). Diagnosis-related frequency of compulsory measures in 10 German psychiatric hospitals and correlates with hospital characteristics. Soc Psychiatry Psychiatr Epidemiol.

[17] Husum TL, Bjørngaard JH, Finset A, Ruud T (2010). A cross-sectional prospective study of seclusion, restraint and involuntary medication in acute psychiatric wards: patient, staff and ward characteristics. BMC Health Serv Res.

[18] Lay B, Nordt C, Rössler W (2011). Variation in use of coercive measures in psychiatric hospitals. Eur Psychiatry.

[19] Steinert T, Flammer E (2019). Frequency of coercive measures as a quality indicator for psychiatric hospitals. Nervenarzt..

[20] Larue C, Dumais A, Ahern E, Bernheim E, Mailhot MP (2009). Factors influencing decisions on seclusion and restraint. J Psychiatr Ment Health Nurs.

[21] De Benedictis L, Dumais A, Sieu N, Mailhot MP, Létourneau G, Tran MAM (2011). Staff perceptions and organizational factors as predictors of seclusion and restraint on psychiatric wards. Psychiatr Serv.

[22] Van der Schaaf PS, Dusseldorp E, Keuning FM, Janssen WA, Noorthoorn EO (2013). Impact of the physical environment of psychiatric wards on the use of seclusion. Br J Psychiatry.

[23] Bowers L, Stewart D, Papadopoulos C, Iennaco JD (2013). Correlation between levels of conflict and containment on acute psychiatric wards: the city-128 study. Psychiatr Serv.

[24] Bowers L (2014). Safewards: a new model of conflict and containment on psychiatric wards. J Psychiatr Ment Health Nurs.

[25] Papoulias C, Csipke E, Rose D, McKellar S, Wykes T (2014). The psychiatric ward as a therapeutic space: systematic review. Br J Psychiatry.

[26] Jungfer HA, Schneeberger AR, Borgwardt S, Walter M, Vogel M, Gairing SK (2014). Reduction of seclusion on a hospital-wide level: successful implementation of a less restrictive policy. J Psychiatr Res.

[27] Huber CG, Schneeberger AR, Kowalinski E, Fröhlich D, van Felten S, Walter M (2016). Suicide risk and absconding in psychiatric hospitals with and without open door policies: a 15 year, observational study. Lancet Psychiatry.

[28] Schneeberger AR, Kowalinski E, Fröhlich D, Schröder K, van Felten S, Zinkler M (2017). Aggression and violence in psychiatric hospitals with and without open door policies: a 15-year naturalistic observational study. J Psychiatr Res.

[29] Hochstrasser L, Fröhlich D, Schneeberger AR, Borgwardt S, Lang UE, Stieglitz RD (2018). Long-term reduction of seclusion and forced medication on a hospital-wide level: implementation of an open-door policy over 6 years. Eur Psychiatry.

[30] Pollmächer T, Steinert T (2016). Arbitrary classification of hospital policy regarding open and locked doors. Lancet Psychiatry.

[31] Schreiber LK, Metzger FG, Duncker TA, Fallgatter AJ, Steinert T (2019). Open doors by fair means: study protocol for a 3-year prospective controlled study with a quasi-experimental design towards an open ward policy in acute care units. BMC Psychiatry.

[32] Steinert T, Schreiber L, Metzger FG, Hirsch S (2019). Open doors in psychiatric hospitals. An overview of empirical findings. Nervenarzt..

[33] Adorjan K, Steinert T, Flammer E, Deister A, Koller M, Zinkler M (2017). Coercive measures in German hospitals for psychiatry and psychotherapy. A pilot study by the DGPPN to evaluate a uniform assessment instrument. Nervenarzt..

[34] Flammer E, Steinert T (2019). The case register for coercive measures according to the law on assistance for persons with mental diseases of Baden-Wuerttemberg: conception and first evaluation. Psychiatr Prax.

[35] Köpke S, Mühlhauser I, Gerlach A, Haut A, Haastert B, Möhler R (2012). Effect of a guideline-based multicomponent intervention on use of physical restraints in nursing homes. A randomized controlled trial. JAMA..

[36] Handley M, Bunn F, Goodman C (2017). Dementia-friendly interventions to improve the care of people living with dementia admitted to hospitals: a realistic review. BMJ Open.

[37] Handley M, Bunn F, Goodman C (2019). Supporting general hospital staff to provide dementia sensitive care: a realist evaluation. Int J Nurs Stud.

[38] Livingston G, Huntley J, Sommerlad A, Ames D, Ballard C, Banerjee S, et al. Dementia prevention, intervention, and care: 2020 report of the Lancet. 2020;396(10248):413–46. 10.1016/S0140-6736(20)30367-6. 10.1016/S0140-6736(20)30367-6 PMC7392084 32738937

[39] Deutsche Gesellschaft für Gerontopsychiatrie und -psychotherapie (DGGPP). Strukturen gerontopsychiatrischer Versorgung. 2007. https://dggpp.de/grundpositionen.htm. Accessed 11 Mar 2021.

[40] Usher K, Baker JA, Holmes C, Stocks B (2009). Clinical decision-making for ‘as needed’ medications in mental health care. J Adv Nurs.

[41] Georgieva I, Mulder CL, Noorthoorn E (2013). Reducing seclusion through involuntary medication: a randomized clinical trial. Psychiatry Res.

[42] Haw C, Sasegbon H, Ismail I, Pushpanathan M (2015). PRN medication: beliefs and practices of psychiatrists and nurses working in PICUs and secure units. J Psychiatr Intensive Care.

[43] Sjöstrand M, Sandman L, Karlsson P, Helgesson G, Eriksson S, Juth N (2015). Ethical deliberations about involuntary treatment: interviews with Swedish psychiatrists. BMC Med Ethics.

[44] Noorthoorn EO, Voskes Y, Janssen WA, Mulder CL, van de Sande R, Nijman HLI (2016). Seclusion reduction in Dutch mental health care: did hospitals meet goals?. Psychiatr Serv.

[45] Luciano M, De Rosa C, Sampogna G, Del Vecchio V, Giallonardo V, Fabrazzo M (2018). How to improve clinical practice on forced medication in psychiatric practice: suggestions from the EUNOMIA European multicentre study. Eur Psychiatry.

[46] Vishnivetsky S, Shoval G, Leibovich V, Giner L, Mitrany M, Cohen D (2013). Seclusion room vs. physical restraint in an adolescent inpatient setting: patients’ attitudes. Isr J Psychiatry Relat Sci.

[47] Steinert T, Birk M, Flammer E, Bergk J (2013). Subjective distress after seclusion or mechanical restraint: one-year follow-up of a randomized controlled study. Psychiatr Serv.

[48] Chieze M, Hurst S, Kaiser S, Sentissi O (2019). Effects of seclusion and restraint in adult psychiatry: a systematic review. Front Psychiatry.

[49] Gleerup CS, Ostergaard SD, Hjuler RS (2019). Seclusion versus mechanical restraint in psychiatry – a systematic review. Acta Neuropsychiatr.

[50] Georgieva I, Mulder CL, Whittington R (2012). Evaluation of behavioral changes and subjective distress after exposure to coercive inpatient interventions. BMC Psychiatry.

[51] Happell B, Harrow A (2010). Nurses’ attitudes to the use of seclusion: a review of the literature. Int J Ment Health Nurs.

[52] Husum TL, Bjørngaard JH, Finset A, Ruud T (2011). Staff attitudes and thoughts about the use of coercion in acute psychiatric wards. Soc Psychiatry Psychiatr Epidemiol.

[53] Yang CPP, Hargreaves WA, Bostrom A (2014). Association of empathy of nursing staff with reduction of seclusion and restraint in psychiatric inpatient care. Psychiatr Serv.

[54] Aasland OG, Husum TL, Førde R, Pedersen R (2018). Between authoritarian and dialogical approaches: attitudes and opinions on coercion among professionals in mental health and addiction care in Norway. Int J Law Psychiatry.

[55] Hotzy F, Jaeger M, Buehler E, Moetteli S, Klein G, Beeri S (2019). Attitudinal variance among patients, next of kin and health care professionals towards the use of containment measures in three psychiatric hospitals in Switzerland. BMC Psychiatry.

[56] Bigwood S, Crowe M (2008). ‘It’s part of the job, but it spoils the job’: a phenomenological study of physical restraint. Int J Ment Health Nurs.

[57] Moran A, Cocoman A, Scott PA, Matthews A, Staniuliene V, Valimaki M (2009). Restraint and seclusion: a distressing treatment option?. J Psychiatr Ment Health Nurs.

[58] Boumans CE, Egger JIM, Souren PM, Mann-Poll PS, Hutschemaekers GJM (2012). Nurses’ decision on seclusion: patient characteristics, contextual factors and reflexivity in teams. J Psychiatr Ment Health Nurs.

[59] Boumans CE, Egger JIM, Bouts RA, Hutschemaekers GJM (2015). Seclusion and the importance of contextual factors: an innovation project revisited. Int J Law Psychiatry.

[60] D’Ettorre G, Pellicani V (2017). Workplace violence toward mental healthcare workers employed in psychiatric wards. Saf Health Work.

[61] Flammer E, Steinert T, Eisele F, Bergk J, Uhlmann C (2013). Who is subjected to coercive measures as a psychiatric inpatient? A multi-level analysis. Clin Pract Epidemiol Ment Health.

[62] Simpson SA, Joesch JM, West II, Pasic J (2014). Risk for physical restraint or seclusion in the psychiatric emergency service (PES). Gen Hosp Psychiatry.

